# Metformin attenuates fructose-induced pulmonary fibrosis, possibly through the involvement of the TRPC6 channel

**DOI:** 10.1590/1414-431X2026e15255

**Published:** 2026-03-09

**Authors:** B. Serez Kaya, O. Öner, D. Ercetin, M.A. Aydın, E. Akbas, M. Sapmaz Metin, O. Kaya

**Affiliations:** 1 Department of Chest Disease, Faculty of Medicine, Trakya University, Edirne, Turkey; 2 Department of Physiology, Faculty of Medicine, Istanbul Medeniyet University, Istanbul, Turkey; 3 Department of Histology & Embryology, Faculty of Medicine, Trakya University, Edirne, Turkey; 4 Department of Physiology, Faculty of Medicine, Trakya University, Edirne, Turkey; 5 Department of Physiology, Faculty of Medicine, Istanbul Beykent University, Istanbul, Turkey

**Keywords:** Cation channel, Collagen deposition, High fructose diet, Lung injury

## Abstract

Pulmonary fibrosis is a chronic, progressive, fatal lung disease characterized by abnormal lung tissue repair and intense collagen accumulation in the lungs. Despite intensive efforts, no specific treatment has been found. The aim of this study was to investigate the effects of a high-fructose diet on lung tissue, whether the use of metformin corrects the damage caused by high fructose, and the possible relationship with transient receptor potential (TRP) channels. Lung damage was induced by adding 200 g/L fructose to the drinking water of Sprague-Dawley rats (n=32) for 10 weeks. In the groups in which the effect of metformin was examined, metformin (dissolved in saline) was administered intraperitoneally at a dose of 100 mg/kg in the last two weeks of the study. Inflammation and TRP channel levels were measured by ELISA. Histopathological evaluation was also assessed by Masson's Trichrome staining. A high-fructose diet caused collagen deposition, inflammation, and intra-alveolar hemorrhage in lung tissue. Interleukin-6 and TRPC6 channel protein levels in lung tissue were higher in the high-fructose group than in the control group. These effects were prevented by metformin treatment. Metformin administration and manipulation of TRPC6 channels may be a therapeutic target for the treatment of pulmonary fibrosis.

## Introduction

Pulmonary fibrosis is a chronic progressive lung disease with no definitive cure, leading to excessive extracellular matrix deposition in the interstitium, impaired gas exchange, and respiratory failure. The development of pulmonary fibrosis is often preceded by acute lung inflammation, which does not resolve over time and leads to the accumulation of fibrotic tissue in the lungs and respiratory dysfunction. Pulmonary fibrosis is not a single disease, and interstitial lung disease (ILD) and idiopathic pulmonary fibrosis (IPF) are the forms with the worst prognosis, characterized by progressive fibrosis (1). The incidence of IPF is estimated to range from 0.09 to 1.30 per 10,000 people globally (1,2). The therapeutic effects of the two approved drugs for IPF (nintedanib and pirfenidone) are limited, and median survival in IPF patients varies between 2 and 5 years (1,3).

Fructose is a monosaccharide found in fruits, vegetables, honey, and as a component of the disaccharide sucrose. However, the main source of fructose in modern diets is high-fructose corn syrup, which is used as a sweetener in various foods (4). Studies in recent years have shown that consumption of high-fructose drinks is associated with the risk of asthma, chronic bronchitis, and emphysema. In these studies, neutrophil infiltration, increased levels of inflammatory cytokines, and collagen deposition emerged as important factors (5). A recent study demonstrated that high fructose promotes the fibrotic phenotype of human lung epithelial cells (6).

Metformin is an antidiabetic agent. It reduces the amount of glucose produced by the liver and increases peripheral glucose utilization by sensitizing cells to insulin (7). Recent studies have shown that metformin exerts beneficial effects in bleomycin-induced pulmonary fibrosis by acting on several intracellular pathways (8,9). However, its effects on high fructose-induced changes in the lungs are unknown.

Transient receptor potential (TRP) channels, which are expressed in different cells of the respiratory tract, are important for cellular ion homeostasis. They have been shown to play a role in diseases such as cystic fibrosis, asthma, chronic obstructive pulmonary diseases, and pulmonary edema (10). To our knowledge, no study has investigated the role of TRP channels in the effects of high fructose on the lungs.

Although IPF is a condition with high mortality and attracts attention, few studies have examined the possible effects of a fructose-rich diet on lung inflammatory status and pulmonary fibrosis. In this study, we aimed to investigate the changes that a fructose-rich diet causes in lung tissue and the relationship between these changes and TRP channels. We also sought to determine whether metformin use improves the changes caused by high fructose.

## Material and Methods

A total of 32 female Sprague-Dawley rats (13 weeks of age) were divided into four groups (n=8 per group). Animals were kept under laboratory conditions of a 12-h light/dark cycle, 22±2°C, and 40% humidity. All groups were fed *ad libitum* with standard pellet feed for 10 weeks. Measures were taken to minimize discomfort, and the Animal Research Ethics Committee of Trakya University approved all experimental protocols of this study (2023.10.07).

### Animal groups

Control and metformin groups were given normal drinking water, and Fructose and Fructose+Metformin groups were given drinking water and fructose (200 g/L) *ad libitum* for 10 weeks. The fructose dosage was determined based on previous studies conducted in our department (11). During the last two weeks of the 10-week total experimental period, vehicle (saline) was given intraperitoneally to Control and Fructose groups, and 100 mg/kg metformin (dissolved in saline) was given to Metformin and Fructose+Metformin groups. At the end of the 10-week feeding period, all animals were euthanized under general anesthesia (10 mg/kg xylazine and 50 mg/kg ketamine). Following induction of general anesthesia, the lungs of all animals were harvested for analysis. The left lung was placed in 10% formaldehyde solution for pathological examination, and the right lung was washed in cold phosphate buffer and stored at -80°C until analyses were performed.

### Evaluation of inflammation and TRP channels

Interleukin 6 (IL-6), tumor necrosis factor alpha (TNFα), transient receptor potential vanilloid 1 (TRPV1), transient receptor potential vanilloid 4 (TRPV4), and transient receptor potential canonical 6 (TRPC6) levels were measured in lung tissues homogenized in phosphate buffer with commercial ELISA kits (BT LAB, China; E0135Ra, E0764Ra, E1568Ra, E2291Ra, and E1350Ra, respectively). Protein levels in lung tissue were determined by Lowry's method, which is based on complexation of alkaline copper tartrate reagent with peptide bonds (12).

### Histopathology

Lung tissues were fixed in 10% neutral formaldehyde, embedded in paraffin, and cut into 5-μm-thick sections. Masson's Trichrome staining was performed on the sections. Slides were analyzed using light microscopy (Olympus BX51, Japan) at ×200 and ×400 magnifications. Lung tissues were evaluated by an investigator who was blinded to the identity of the slides. Histopathological evaluation was performed on 2 lung tissue sections from each animal, at three random sites per section. Fibrosis caused by collagen deposition in the lung was demonstrated with Masson's Trichrome staining according to the Ashcroft score (13,14).

In addition, the extent of histopathological lesions was evaluated using a score table graded from 1 to 6 and the evaluation criteria of Yang et al. (15).

### Statistical analysis

The results are reported as means±SD. The suitability of study data to normal distribution was verified with the Shapiro-Wilk test, and the homogeneity of variances was verified with the Levene test. Parametric test conditions were not met for histologic lesion score and collagen deposition data. Kruskal-Wallis test and Dunnett's T3 test were used as *post hoc* analyses for these parameters. One-way analysis of variance (ANOVA) was used to compare means of other data, and Tukey's HSD test was used for *post hoc* analyses of between-group comparisons. Statistical significance was set as a P value lower than 0.05.

## Results

### Inflammation markers

**Figure 1:**
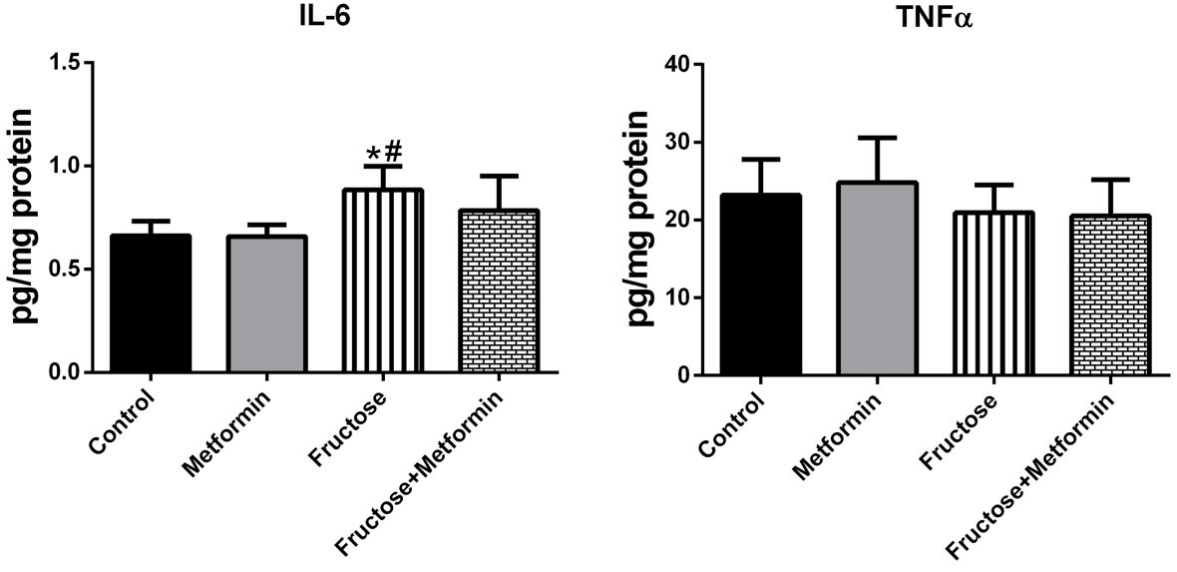
Inflammation markers in lung tissue samples. Data are reported as means±SD, n=8. *P<0.05 compared to the Control group; ^#^P<0.05 compared to the Metformin group (ANOVA and *post hoc* Tukey test). IL: interleukin; TNFα: tumor necrosis factor alpha.

Inflammation markers in lung tissue samples are shown in Figure 1. IL-6 level was significantly higher in the Fructose group (0.88±0.11) than in the Control (0.66±0.07) and Metformin groups (0.65±0.05). IL-6 levels were similar in the Control and Fructose+Metformin groups. TNFα levels measured to evaluate inflammation were similar in all study groups.

### TRP channels

**Figure 2:**
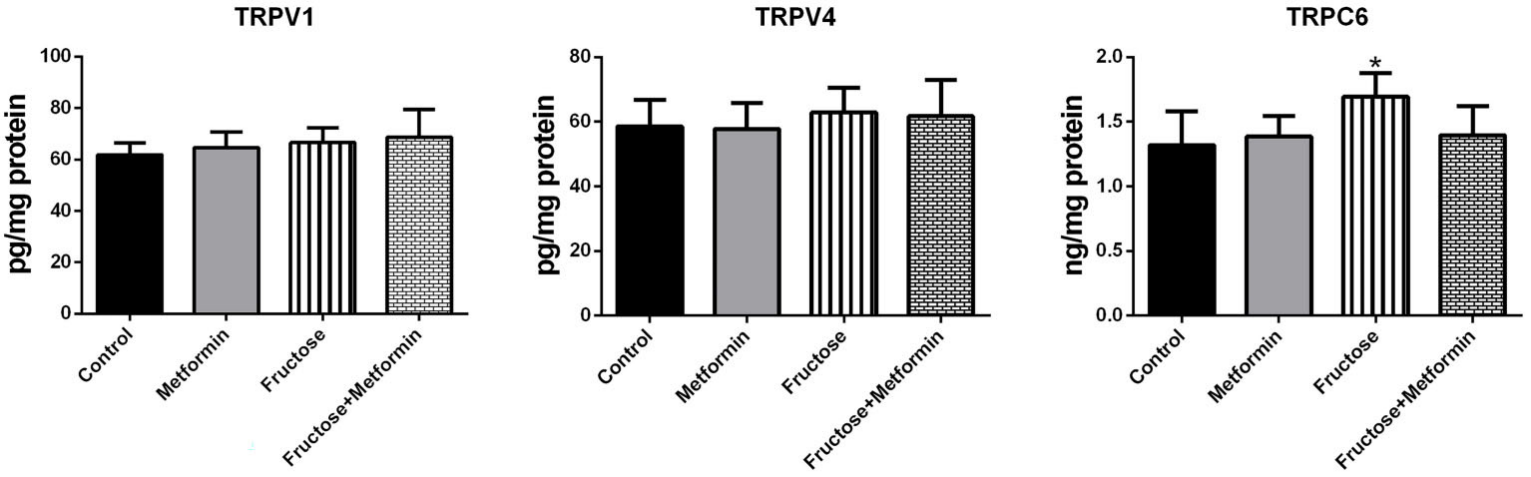
Transient receptor potential (TRP) channels in lung tissue samples. Results are reported as means±SD, n=8. *P<0.05 compared to the Control group (ANOVA and *post hoc* Tukey test).

To study a potential role of cation channels in lung injury, we analyzed TRPV1, TRPV4, and TRPC6 levels in lung tissue samples. As demonstrated in Figure 2, the level of TRPC6 was higher in the Fructose group than in the Control group [1.69±0.18 *vs* 1.31±0.26 ng/mg protein (P=0.04)]. TRPC6 levels were similar among the other groups. TRPV1 and TRPV4 levels were also similar among the groups.

### Histopathological findings

**Figure 3:**
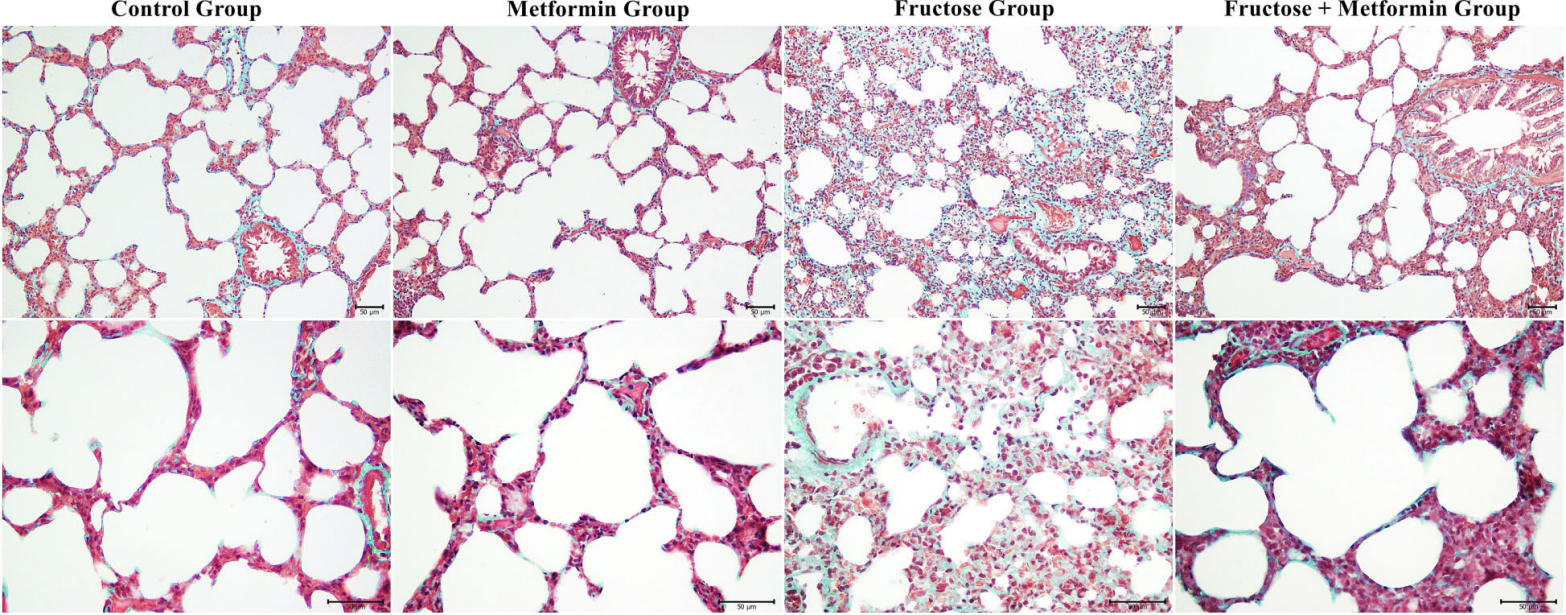
Lung histopathology and collagen deposition of all groups. Masson's Trichrome staining; scale bar 50 μm.

**Figure 4:**
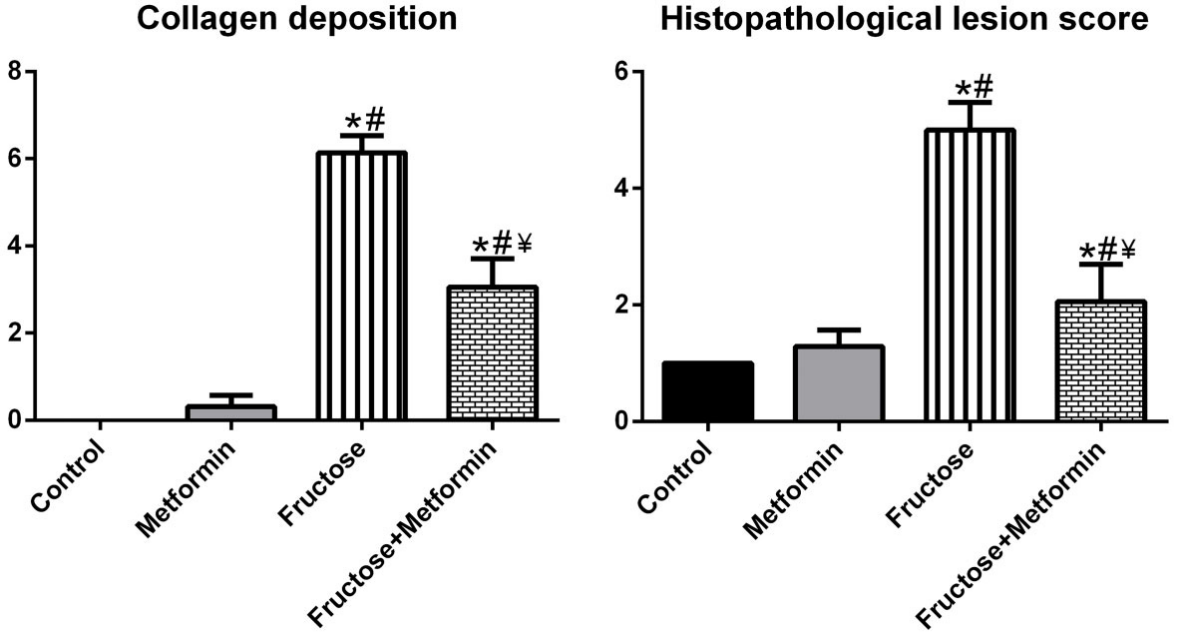
Morphological analysis of the groups. Data are reported as means±SD, n=8. *P<0.05 compared to the Control group; ^#^P<0.05 compared to the Metformin group; ^¥^P<0.05 compared to the Fructose group. Kruskal-Wallis test and *post hoc* Dunnett's T3 test.

Normal histological structure was observed in the lung tissue samples of the Control group. Collagen deposition in the Metformin group was similar to that in the Control group. Masson-stained lung tissue showed massive collagen deposition in the pulmonary septum (green color by light green staining) in the fructose-treated groups, but metformin reduced collagen deposition (Figure 3). No histopathological changes were observed in the Control group. The lung injury scores, including alveolar congestion, inflammatory cell infiltration, and intra-alveolar hemorrhage, were higher in lung tissue sections from the Fructose group than the Control and Metformin groups. The morphological score analysis of the groups is shown in Figure 4.

## Discussion

The main finding of this study was that a high-fructose diet caused collagen accumulation, inflammation, and intra-alveolar hemorrhage in lung tissue and that these effects were prevented by metformin treatment. In addition, TRPC6 channel protein levels were found to be higher in the lung tissue of the high-fructose group. To our knowledge, this is the first study to investigate the relationship between lung injury and TRP channels in high-fructose fed rats.

Despite much attention, we still know less than we should about the mechanisms underlying pulmonary fibrosis. Although the underlying mechanisms are not clear, it is accepted that diet is an important factor that can alter lung structure and function. In a study of mice fed a diet containing 20% fructose, destruction and remodeling of the lung parenchyma was observed and respiratory mechanics were found to be impaired (5). These changes in the lungs were found to be associated with inflammatory cytokine levels. In another study conducted on mice receiving a diet containing 10% fructose, inflammatory cytokine levels were found to be similar to the control group in the group receiving high fructose, while they were higher in the group receiving high fructose along with a high-fat diet (16). Data from a prospective cohort study showed that adherence to healthy dietary recommendations was associated with milder chronic inflammation as assessed by serum IL-6. Long-term non-compliance with healthy dietary recommendations resulted in higher levels of IL-6 (17).

In this study, the IL-6 level in the lung tissues of high fructose-fed rats was higher than the Control group, while the TNFα level was similar between the groups. Our results suggest that a high-fructose diet may play a role in the pathogenesis of fibrosis by causing low-grade but persistent inflammation. It has been observed that in pulmonary fibrosis, damage to the lung is repaired abnormally and fibrosis then progresses with the contribution of epithelial cells, fibroblasts, and myofibroblasts.

The TRPC6 channel is expressed in airway smooth muscle cells and contributes to the pathogenesis of lung diseases such as cystic fibrosis, chronic obstructive pulmonary disease (COPD), asthma, pulmonary edema, and pulmonary fibrosis (18-21). A study in a bleomycin-induced pulmonary fibrosis model reported that TRPC6 is responsible for increased vascular permeability in the lung, which may facilitate the migration of circulating fibrocytes to injured areas. In the same study, it was reported that TRPC6-deficient lungs showed less severe pulmonary fibrosis than wild-type lungs. It has also been suggested that TRPC6 may be an important biomarker for myofibroblast differentiation (22). To our knowledge, our study is the first to assess TRPC6 levels in the lungs of rats fed high fructose. Our data showed that TRPC6 levels were high in the fructose-induced fibrosis model, similar to those in bleomycin-induced fibrosis model. In a recent study of IPF patients, TRPV1 receptors were shown to be similar to controls at the gene and protein levels (23). In a study conducted in guinea pigs, TRPV1 expression was upregulated in the lung tissue of bleomycin-treated animals (24). In another study, TRPV4-deficient mice were protected from the fibrotic effects of bleomycin. In addition, TRPV4 blockade prevented myofibroblast differentiation (25). In our study, TRPV1 and TRPV4 levels were similar in all groups. The discrepancy in study results may be due to differences in the species and models used.

Metformin is a pharmacological agent that inhibits gluconeogenesis, increases peripheral glucose utilization by sensitizing cells to insulin and, for these reasons, is widely used to lower blood glucose in type II diabetics. Previous studies have shown that metformin attenuates pulmonary fibrosis through activation of AMP-activated protein kinase (AMPK), modulation of the nicotinamide adenine dinucleotide phosphate oxidase 4 NOX4, transforming growth factor beta (TGF-β) and AMPK/FOXM1 signaling pathways (8,26,27). In a recent study, metformin inhibited collagen production in primary human lung fibroblasts and *ex vivo* cultured human IPF precision-cut lung slices and increased myo-lipofibroblast transdifferentiation. In the *in vivo* part of the study, therapeutic use of metformin in the bleomycin-induced pulmonary fibrosis model in mice resulted in accelerated resolution of fibrosis by inducing lipogenic differentiation of myofibroblasts (7). In our study, consistent with the aforementioned studies, metformin administration prevented high-fructose diet-induced fibrotic changes in the lung. In addition, the lung injury score, which includes alveolar congestion, inflammatory cell infiltration, and intra-alveolar bleeding, was lower in the Metformin group than in the high-fructose group. Man et al. (28), in a systematic review examining studies published from 2018 to 2023, compared the therapeutic efficacy of nintedanib and pirfenidone in IPF. Their analysis demonstrated that both antifibrotic agents slow the rate of decline in lung functions relative to untreated individuals, with minimal differences in effectiveness between the two medications. Overall, nintedanib and pirfenidone are reported to be safe and generally well-tolerated. Nevertheless, no available therapy has yet been shown to repair established lung damage or reverse the progressive fibrotic remodeling that characterizes IPF. Although metformin is not currently an approved antifibrotic therapy, its ability to modulate inflammatory pathways and TRP-channel expression places it within a mechanistic spectrum that partially overlaps with these drugs. While our study did not perform head-to-head comparisons, the observed alteration in inflammatory markers suggests that metformin may exert complementary or additive effects to existing treatments. Future comparative or combination studies will be essential to define metformin's relative effect size and potential role within antifibrotic therapeutic strategies.

Our study provides an initial framework describing alterations in TRP channels in pulmonary fibrosis and highlighting their potential pathophysiological relevance. A key limitation of this study was the absence of functional assays to directly validate TRPC6 involvement. To clarify whether TRPC6 contributes directly to the observed responses, future investigations would benefit from targeted mechanistic approaches, including the use of TRPC6 agonists or antagonists, siRNA-mediated knockdown, or selective pharmacological blockade. These strategies will be essential for determining whether TRPC6 exerts a causal influence in this context. Our study primarily focused on evaluating the initial cytokine profile associated with metformin administration, and IL-6 and TNF-α were selected as representative early-phase inflammatory markers based on previous literature. In future studies, assessment of downstream or complementary mediators - such as TGF-β1, IL-1β, MCP-1, and NF-κB activity - will be important for deepening mechanistic insight, particularly given their close association with fibrotic pathways that were not examined in the present research. In our study, we performed quantitative measurements of protein levels using ELISA. Complementary techniques such as western blotting or immunohistochemistry were beyond the resources for the current study. Such complementary techniques will provide additional confirmation of protein localization and cellular origin.

In conclusion, metformin treatment reduced lung collagen deposition and damage score in rats fed a high-fructose diet. We have found that TRPC6 channels may play a role in pulmonary fibrosis caused by a high-fructose diet. To our knowledge, this is the first study to investigate the relationship between lung damage caused by a high-fructose diet and the TRP channel. It would be useful to conduct more detailed studies to determine the molecular mechanisms and cellular pathways through which TRP channels are involved in high-fructose diet-induced pulmonary fibrosis. This will help to determine whether TRP channel activity and metformin treatment can be therapeutic targets in the prevention of fructose-induced pulmonary fibrosis.

## Data Availability

The datasets generated and/or analyzed during the current study are available from the corresponding author on reasonable request.
